# Efficacy and Safety of Anti-calcitonin Gene-Related Peptide (CGRP) Monoclonal Antibodies in Preventing Migraines: A Systematic Review

**DOI:** 10.7759/cureus.45560

**Published:** 2023-09-19

**Authors:** Meghana Reddy Muddam, Omobolanle A Obajeun, Abdelrahman Abaza, Arturo P Jaramillo, Faten Sid Idris, Humna Anis Shaikh, Ilma Vahora, Kiran Prasad Moparthi, Majdah T Al Rushaidi, Tuheen Sankar Nath

**Affiliations:** 1 General Practice, California Institute of Behavioral Neurosciences & Psychology, Fairfield, USA; 2 Pediatrics, California Institute of Behavioral Neurosciences & Psychology, Fairfield, USA; 3 Pathology, California Institute of Behavioral Neurosciences & Psychology, Fairfield, USA; 4 General Surgery, California Institute of Behavioral Neurosciences & Psychology, Fairfield, USA; 5 Psychology, California Institute of Behavioral Neurosciences & Psychology, Fairfield, USA; 6 Surgical Oncology, California Institute of Behavioral Neurosciences & Psychology, Fairfield, USA

**Keywords:** migraine treatment, patient characteristics, safety, safety and efficacy, anti calcitonin gene-related peptide (cgrp) monoclonal antibodies, cgrp, migraine

## Abstract

The neuropeptide calcitonin gene-related peptide (CGRP) is an essential pathophysiological treatment for migraines. A unique class of medications called CGRP monoclonal antibodies target CGRP and its receptor and have demonstrated promising benefits in the treatment and prevention of migraines. This study sought to identify and assess the quality of existing systematic reviews about the effectiveness of CGRP antibodies for preventing migraines, as well as systematically review and synthesize the evidence on these topics. This included the four Food and Drug Administration (FDA)-approved medications erenumab, galcanezumab, fremanezumab, and eptinezumab. The effectiveness and safety of these monoclonal antibodies in preventing migraines should also be examined in light of patient characteristics, and any gaps in the body of knowledge should be noted in order to suggest new lines of investigation.

Data gathering included a thorough search of internet databases (PubMed, Cochrane Library, Web of Science, and Scopus) for relevant research released between 2018 and 2023. The findings imply that CGRP monoclonal antibodies are efficient and secure for preventing migraines and may be considered a first-line alternative for treating migraines and drug misuse. The results further imply that combination treatment with CGRP antibodies and onabotulinumtoxinA may enhance the prevention of migraine in adults. With suggestions for more studies to find and address these variables, the significance of genetic and epigenetic factors in the progression of pediatric patients' acute postoperative pain to chronic postsurgical pain is underlined. All four anti-CGRP monoclonal antibodies, erenumab, fremanezumab, galcanezumab, and eptinezumab, were shown to be safe and effective for the prevention of migraine when the research additionally looked at their individual effectiveness and safety. Additionally, the study discovered considerable variances in effectiveness amongst various groups. However, further investigation is required to establish the best time and dosage and the effect of patient characteristics on the effectiveness and safety of these medications.

## Introduction and background

There are about one billion people worldwide who suffer from migraine, a common neurological condition [1]. Recurrent headaches, often accompanied by vomiting, nausea, and sensitivity to sound and light, were defined as this incapacitating syndrome [2]. Migraine headaches can be severe, and in some cases, they can last for days [3]. The environment, genetics, and lifestyle were the main factors that played a vital role in the development of migraine pain [4]. Calcitonin gene-related peptide (CGRP) is a neuropeptide that is produced by both peripheral and central neurons [5,6]. It affects a number of physiological processes, such as inflammation, vasodilation, and pain perception [7]. According to studies, CGRP levels are raised in migraineurs when they are having an episode [8]. Due to this, CGRP monoclonal antibodies (mAbs) have been developed as a therapeutic target for the avoidance of migraines [9]. These mAbs bind to CGRP or its receptor, blocking their action and preventing the onset of migraine attacks [10].

Recent years have seen a major increase in interest in the use of CGRP mAbs in migraine prophylaxis [11]. Erenumab, fremanezumab, galcanezumab, and eptinezumab are the main drugs involved in the prevention and treatment of migraine [12]. These medications have been shown to be successful in lowering the frequency and intensity of migraine episodes as well as enhancing the quality of life for migraine sufferers [13]. The FDA first authorized a CGRP mAb in 2018 with erenumab [13]. The CGRP receptor is the target of this human monoclonal antibody [11]. In around 40%-50% of migraine sufferers, clinical studies have shown that erenumab lowers the frequency of monthly migraine days by 50% or more [14]. It has also been shown to be well-tolerated, with few adverse effects [14]. Fremanezumab and galcanezumab are humanized monoclonal antibodies that target CGRP itself [15]. Clinical trials have shown that these drugs reduce the number of patients suffering from migraines by up to 50% or more in approximately 50%-60% of individuals with migraines [16]. They have also been shown to be well-tolerated, with few adverse effects [15].

Eptinezumab is the newest CGRP mAb to be approved by the FDA in 2020. Clinical trials have shown that eptinezumab reduces the intensity of migraines by up to 50% or more in approximately 30%-40% of individuals with migraines [14]. It has also been shown to be well-tolerated, with few adverse effects [17]. In terms of safety, CGRP mAbs have been generally well-tolerated [18]. Given that CGRP serves a number of physiological tasks, there have been questions over the possible long-term implications of inhibiting it [19]. To completely assess the safety profile of these medications, long-term trials are required [20]. These medications have been shown to be successful in lowering the frequency and intensity of migraine episodes and enhancing the quality of life for migraine sufferers [21, 22]. Despite these limitations, the use of CGRP mAbs represents a significant advancement in the field of migraine treatment and offers new hope for individuals with this debilitating condition [23-25]. This study sought to identify and assess the quality of existing systematic reviews about the effectiveness of CGRP antibodies for preventing migraines, as well as systematically review and synthesize the evidence on these topics.

This study which identifies and evaluates the quality of existing systematic reviews on the role of CGRP antibodies in preventing migraine is critical for providing clinicians and patients with the most up-to-date and reliable information to make informed decisions about treatment options. With several drugs currently approved by the FDA, it is essential to have a thorough understanding of their efficacy and safety profiles, as well as any potential limitations or adverse effects. Systematically reviewing and synthesizing the available evidence on the role of CGRP monoclonal antibodies in preventing migraine, including the four FDA-approved drugs, can help identify any gaps in the literature and highlight areas where additional research is needed. By examining the impact of patient characteristics, such as age, sex, and comorbidities, on the efficacy and safety of these drugs, clinicians can better tailor treatment plans to individual patient needs and improve patient outcomes. By identifying gaps in the existing literature and proposing future research directions, this study can help guide the development of new research studies and contribute to the advancement of migraine treatment.

This study assesses the effectiveness and safety of CGRP monoclonal antibodies for migraine prevention and treatment. It examines how patient factors impact antibody efficacy and safety. The study also identifies knowledge gaps about optimal timing, dosage, and patient selection criteria for these antibodies. Reviewing existing research, it aims to provide insights into using CGRP antibodies as a first-line treatment option for migraines based on their therapeutic potential and patient-specific suitability.

## Review

Materials and methods

We used Preferred Reporting Items for Systematic Reviews and Meta-Analysis (PRISMA) for conducting this systematic review.

Search Strategy and Inclusion Criteria

The data for this study was collected from different databases, including PubMed, Cochrane Library, Web of Science, and Scopus. The search included content from publications between 2018 and 2023. The search phrases included "migraine prevention," "CGRP monoclonal antibodies," "erenumab," "fremanezumab," "galcanezumab," and "eptinezumab." Related research articles or papers published in English from 2018 to 2023 were included in this research study. Only those articles and research papers that were published after 2018 and directly relevant to the CGRP monoclonal antibodies in the treatment of migraine are included in the exclusion criteria.

Data Extraction and Quality Assessment

The data were collected using different electronic databases like Embase, Cochrane Library, Scopus, Web of Science, and PubMed. The search included articles published from 2018 to 2023. The data included study design, sample size, patient characteristics, intervention details, and outcomes. The Risk Of Bias In Non-randomised Studies - of Interventions (ROBINS-I) tool was used for non-randomized studies.

Quality of Data Collection

A thorough search of PubMed and other electronic databases was conducted to find relevant data related to reviews of studies published between 2018 and 2023. The keyword list for the search strategy includes phrases like "CGRP", "monoclonal antibodies," "migraine," erenumab," "fremanezumab," "galcanezumab," "eptinezumab," "systematic review," and "meta-analysis [26]. To rate the effectiveness of the included systematic reviews, we utilized the A Measurement Tool to Assess Systematic Reviews, version 2' (AMSTAR 2) tool. Table 1 shows the preferred tools for assessment.

**Table 1:** Quality assessment using the preferred tools JBI: Joanna Briggs Institute; AMSTAR: A Measurement Tool to Assess Systematic Reviews; PRISMA: Preferred Reporting Items for Systematic Reviews and Meta-Analyses

Study Types	Tools	Number
Observational studies	JBI tool	5
Systematic review	AMSTAR checklist	1
Meta-analysis	PRISMA guidelines	1
Protocol	Not applicable	1

Table 2 shows quality assessment using the Joanna Briggs Institute (JBI) check tool for observational studies.

**Table 2:** Quality assessment using the Joanna Briggs Institute (JBI) check tool for observational studies

Year of publication	Were the two groups similar and recruited from the same population?	Were the exposures measured similarly to assign people to both exposed and unexposed groups?	Was the exposure measured in a valid and reliable way?	Where confounding factors identified?	Were the strategies to deal with confounding factors stated?	Were the groups/participants free of the outcome at the start of the study (or at the time of exposure)?	Were the outcomes measured in a valid and reliable way?	Was the follow-up time reported sufficient to be long enough for outcomes to occur?	Was the follow-up complete, and if not, were the reasons for loss to follow-up described and explored?	Were strategies to address incomplete follow-up utilized?	Was appropriate statistical analysis used?
Tepper et al., 2019 [27]	Yes	Yes	Yes	Yes	Yes	Yes	Yes	Yes	Yes	Yes	Yes
Mechtler et al., 2022 [21]	-	-	-	-	-	-	-	-	-	-	-
Gklinos et al., 2021 [28]	-	-	-	-	-	-	-	-	-	N/A	-
Wang et al., 2021 [20]	-	-	-	-	-	-	-	-	-	-	Yes
Vandervorst et al., 2011 [6]	­-	-­	-­	-­	-­	-­	-­	-­	-­	-	-
Fernández-Bravo- Rodrigo et al., 2022 [15]	­-	­-	­-	­-	­-	­-	­-	­-	­-	-	

Results

Search Outcomes

**Figure 1:**
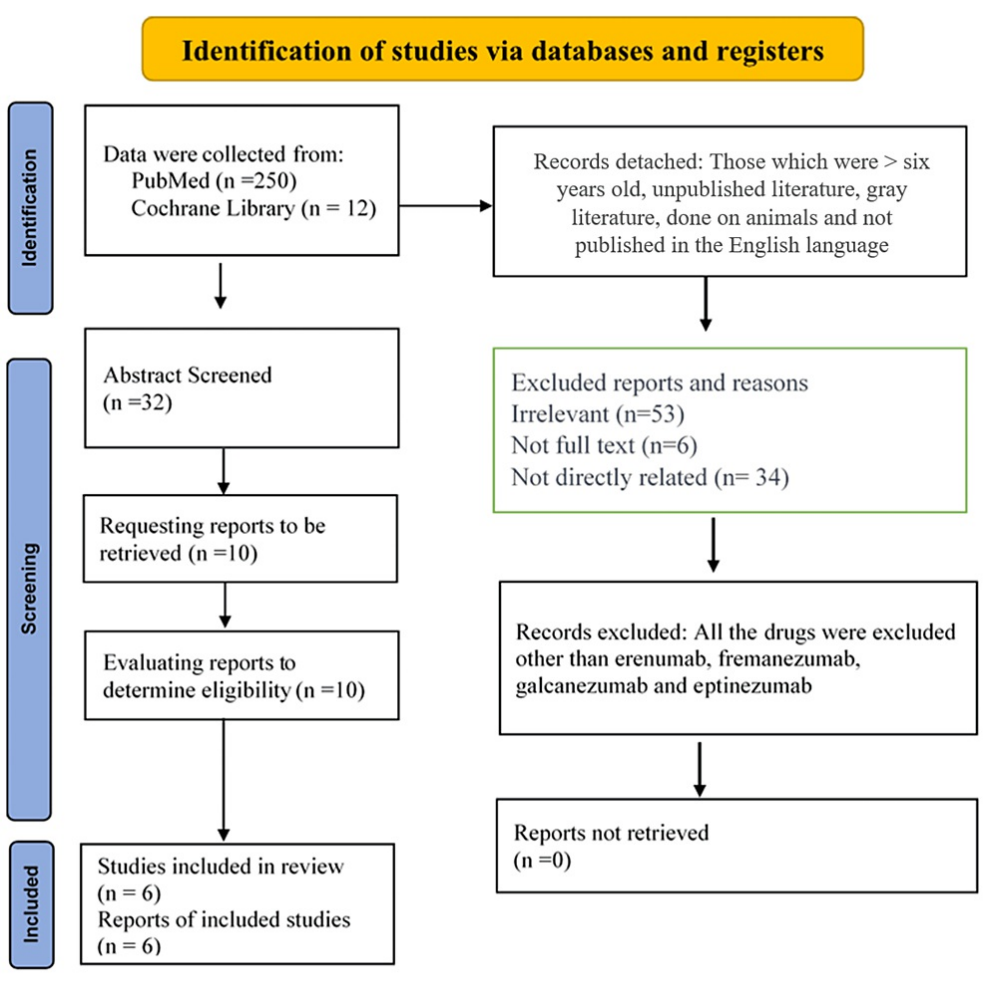
The article filtering processes are depicted in the PRISMA flow diagram. PRISMA: Preferred Reporting Items for Systematic Reviews and Meta-Analyses

In this study, 250 research articles were selected for review using PubMed from different databases. The selection of the papers was based on eligibility criteria (inclusion and exclusion), including relevance (monoclonal antibodies and migraine), language, and defined drugs (erenumab, fremanezumab, galcanezumab, and eptinezumab). The search included articles published from 2018 to 2023. The search terms included "CGRP antibodies," "migraine prevention," "erenumab," "fremanezumab," "galcanezumab," and "eptinezumab." The inclusion criteria included articles published between 2018 and 2023 and directly related to the treatment and prevention of migraine. The study population consisted of adults with a history of migraine. While the exclusion criteria include articles published before 2018, they are not directly related to the efficacy and safety of CGRP monoclonal antibodies in preventing migraine [26]. All the irrelevant papers were excluded from the list, and finally, eight papers (two systematic reviews and six observational studies) were selected for final review. This study was conducted to identify and evaluate the quality of existing systematic reviews on the treatment and prevention of migraine. Figure 1 shows the PRISMA flow diagram of the article filtering process.

Table 3 presents a collection of studies conducted by different authors over various years, each addressing different aspects of migraine prevention and pain management and contributing to our understanding of different treatment approaches and factors influencing migraine and pain management.

**Table 3:** The efficacy and safety of anti-CGRP monoclonal antibodies in preventing migraine CGRP: calcitonin gene-related peptide; RCTs: randomized controlled trials; AMSTAR: a measurement tool to assess systematic reviews; N/A: not applicable

Study ID	Author	Publication year	Type of study	Main Objectives	Method used	Conclusion and recommendations	AMSTAR 2 score
1	Tepper SJ et al. [27]	2019	Randomized trial subgroup analysis	Assessment of the effectiveness of erenumab for the treatment of migraine	Randomized trial	Erenumab may be an effective treatment option for patients with chronic migraines and medication overuse.	10/16
2	Mechtler et al. [21]	2022	Real-world evidence	To evaluate the safety and efficacy of CGRP monoclonal antibody therapy added to onabotulinumtoxinA treatment for migraine prevention	Observational study	Combination therapy may be a promising approach for migraine prevention	9/16
3	Gklinos et al. [28]	2021	Meta-analysis	To evaluate the efficacy and safety of monoclonal antibodies targeting CGRP or its receptor for migraine prophylaxis	Meta-analysis of RCTs	Monoclonal antibodies may be considered as a first-line treatment for migraine prevention	8/16
4	Vandervorst et al. [6]	2021	Systematic review and meta-analysis	To assess the effect of CGRP monoclonal antibodies in migraine prevention	Meta-analysis of RCTs	CGRP monoclonal antibodies may be a promising treatment option for patients with migraine	11/16
5	Fernández-Bravo-Rodrigo J et al. [15]	2022	Multiple-treatment systematic review and meta-analysis	To evaluate the safety and efficacy of CGRP monoclonal antibodies for the preventive treatment of migraine	Systematic review and meta-analysis of RCTs	CGRP monoclonal antibodies may be considered as a first-line treatment option for migraine prevention	10/16
6	Wang et al. [20]	2021	Meta-analysis	To evaluate the efficacy and safety of monoclonal antibodies targeting CGRP or its receptor in migraine prophylaxis	Meta-analysis of RCTs	Monoclonal antibodies may be considered a promising treatment option for migraine prevention	N/A

Table 4 shows a summary of the available evidence on the efficacy and safety of CGRP monoclonal antibodies in preventing migraine.

**Table 4:** Summarizing the available evidence on the efficacy and safety of CGRP monoclonal antibodies in preventing migraine CGRP: calcitonin gene-related peptide

CGRP monoclonal antibody	Function	Effectiveness	Name and year of studies	Conclusion and recommendations
Erenumab	Binds to CGRP receptor	Effective	Tepper et al., 2019 [27]; Mechtler et al., 2022 [21]; Gklinos et al., 2021 [28]; Lampl et al., 2023 [5]; Fernández-Bravo-Rodrigo et al., 2022 [15]; Chiang and Schwedt, 2020 [18]; Mathews et al., 2023 [19];	Erenumab is effective and well-tolerated for the prevention of migraines, especially in patients with chronic migraines and medication overuse
Fremanezumab	Binds to CGRP	Effective	Mechtler et al., 2022 [21]; Gklinos et al., 2021 [28]; Lampl et al., 2023 [5]; Fernández-Bravo-Rodrigo et al., 2022 [15]; Chiang and Schwedt, 2020 [18]; Mathews et al., 2023 [19];	Fremanezumab is effective and well-tolerated for the prevention of migraines, especially in patients with chronic migraines and medication overuse
Galcanezumab	Binds to CGRP	Effective	Mechtler et al., 2022 [21]; Gklinos et al., 2021 [28]; Lampl et al., 2023 [5]; Fernández-Bravo-Rodrigo et al., 2022 [15]; Chiang and Schwedt, 2020 [18]; Mathews et al., 2023 [19];	Galcanezumab is effective and well-tolerated for the prevention of migraines, especially in patients with chronic migraines and medication overuse
Eptinezumab	Binds to CGRP	Effective	Mechtler et al., 2022 [21]; Gklinos et al., 2021 [28]; Lampl et al., 2023 [5]; Fernández-Bravo-Rodrigo et al., 2022 [15]; Chiang and Schwedt, 2020 [18]; Mathews et al., 2023 [19];	Eptinezumab is effective and well-tolerated for the prevention of migraines, especially in patients with chronic migraines and medication overuse

Discussion

Anti-CGRP Monoclonal Antibodies in Preventing Migraine

Anti-CGRP monoclonal antibodies have emerged as a revolutionary class of medications in the field of migraine prevention [27]. Migraine, a debilitating neurological disorder characterized by severe headache attacks, affects millions of people worldwide [28, 29]. Calcitonin gene-related peptide, a neuropeptide found in the central and peripheral nervous systems, has been implicated in the pathophysiology of migraine [30]. By targeting and inhibiting the activity of CGRP, monoclonal antibodies have shown remarkable efficacy in reducing the frequency, severity, and duration of migraine attacks [31]. This breakthrough therapy offers new hope to individuals suffering from chronic or episodic migraines, providing them with a potential means of achieving a better quality of life [32].

New Era in Migraine Management: The Efficacy of Anti-CGRP Monoclonal Antibodies

Anti-CGRP monoclonal antibodies have emerged as a groundbreaking approach to preventing migraine. Previous studies have focused on various preventive therapies such as beta-blockers, antiepileptic drugs, and antidepressants, but their efficacy has been limited and often accompanied by significant side effects. The advent of anti-CGRP monoclonal antibodies has brought about a new era in migraine management [33]. Clinical trials and real-world studies have consistently demonstrated the effectiveness of these antibodies in reducing the frequency of migraine attacks and improving patients' overall quality of life. A study was reported by Tepper et al. (2019) to investigate the impact of anti-CGRP monoclonal antibodies in preventing migraine [27]. In this study, a total of 667 adult patients with chronic migraines were analyzed by demonstrated erenumab (a human anti-CGRP monoclonal antibody). This study reported a significant decline (≥50%) in the severity and duration of migraines (on a monthly basis) in patients who were treated with erenumab monoclonal antibodies. The findings of this study highlight the potential of anti-CGRP monoclonal antibodies, specifically erenumab, as an effective and well-tolerated treatment option for preventing chronic migraines [27].

Mechtler et al. (2022) conducted a study to investigate the impacts of the CGRP monoclonal antibody (onabotulinumtoxinA) for the treatment and prevention of migraine in adult patients. In this study, the impact of onabotulinumtoxinA and calcitonin anti-CGRP monoclonal antibodies was analyzed. These drugs were provided to the patients with migraines for 12 months. The findings of this study concluded that the onabotulinumtoxinA and calcitonin anti-CGRP monoclonal antibodies were more effective for the treatment of chronic migraine [34]. This study suggests that the combination of onabotulinumtoxinA and CGRP mAbs may offer incremental benefits in reducing monthly headache days (MHD) for patients with migraines [35]. Another study reported by Gklinos et al. (2021) also supports the findings of Mechtler et al. (2022). This study reported that the CGRP monoclonal antibody is a vital drug for the treatment of migraine [36]. This study concludes that these antibodies were the main therapeutic agents for a diverse group of pathophysiological disorders such as multiple sclerosis, migraines, etc. This study concluded that eptinezumab, fremanezumab, and galcanezumab CGRP monoclonal antibodies have primitive impacts on the treatment of migraine in humans. Dourson et al. (2022) revised a study to study the role of CGRP monoclonal antibodies in the treatment of migraine [12]. The findings of this study focus on the genetic and epigenetic mechanisms underlying pediatric chronic postsurgical pain (CPSP) [37]. The findings suggest that animal models can provide insights into genomic mechanisms, such as gene variants and differential DNA methylation, associated with CPSP susceptibility in humans [38].

Vandervorst et al. (2021) analyzed the impacts of CGRP on the treatment and prevention of migraine [6]. The findings demonstrate that CGRP monoclonal antibodies not only show comparable or higher efficacy but also offer robustness in the efficacy signal, as evidenced by multiple large-scale randomized clinical trials. The findings of this study also show their long-term safety, making them a valuable option for migraine prevention. Fernández-Bravo-Rodrigo et al. (2022) also revised a similar study and supported the findings of Vandervorst et al. (2021) [15,6]. This study utilizes meta-analytical methods to provide comprehensive evidence on the efficacy and safety of different CGRP monoclonal antibodies for the treatment of migraine [39]. This study also concluded that CGRP antibodies play a vital role in the prevention and treatment of chronic migraine. Another study with similar findings was reported by Wang et al. (2021) [20]. This study was conducted to compare the effectiveness of different CGRP monoclonal antibodies in adult patients with migraines. In this study, a total of 8,926 patients suffering from migraines were analyzed. The findings of this study concluded that eptinezumab, erenumab, fremanezumab, and galcanezumab significantly reduced monthly migraine days compared to placebo.

In this review study, the anti-CGRP monoclonal antibodies and their role in preventing migraine were analyzed. A total of eight studies were reviewed in this study. The findings from multiple studies demonstrate the effectiveness of anti-CGRP monoclonal antibodies in preventing and treating migraines. Clinical trials and real-world studies consistently show that these antibodies, such as erenumab, eptinezumab, fremanezumab, and galcanezumab, significantly reduce the frequency and severity of migraine attacks. These antibodies offer robust efficacy signals and have shown long-term safety, making them valuable options for migraine prevention. This study concluded that CGRP monoclonal antibodies have emerged as a groundbreaking approach in migraine management, offering an effective and well-tolerated treatment option for both chronic and episodic migraines.

Limitations of the study

This study investigates the role of CGRP monoclonal antibodies in the treatment and prevention of migraine. However, this study has some limitations. In this study, only those research articles that were published between 2018 and 2023, and the articles had the objective of investigating the role of CGRP monoclonal antibodies in preventing migraine were selected. Similarly, only those articles were selected for this study that were published in English.

## Conclusions

This study strongly supports the effectiveness of anti-CGRP monoclonal antibodies in preventing and treating migraines. Clinical trials and real-world studies consistently demonstrate a reduction in migraine frequency and improved quality of life for patients treated with these antibodies, particularly erenumab, eptinezumab, fremanezumab, and galcanezumab. The findings highlight the potential of CGRP monoclonal antibodies as an effective and well-tolerated treatment option for chronic migraine. They offer robust efficacy signals, and long-term safety, and may provide incremental benefits when combined with other therapies. However, further research is needed to determine the optimal timing, dosage, and duration of treatment with these antibodies. Additionally, more studies are required to elucidate how patient characteristics such as age, gender, and comorbidities may impact treatment efficacy and safety. Research into the potential long-term effects and comparative effectiveness of the different CGRP monoclonal antibodies would also help guide clinical decision-making. Further exploration of combination therapies involving CGRP antibodies is another area warranting additional investigation.
